# Additive Manufactured Strain Sensor Using Stereolithography Method with Photopolymer Material

**DOI:** 10.3390/polym15040991

**Published:** 2023-02-16

**Authors:** Ishak Ertugrul, Osman Ulkir, Sezgin Ersoy, Minvydas Ragulskis

**Affiliations:** 1 Department of Mathematical Modelling, Kaunas University of Technology, 44138 Kaunas, Lithuania; 2 Department of Electric and Energy, Mus Alparslan University, 49250 Mus, Turkey; 3 Department of Mechatronic Engineering, Marmara University, 34565 Istanbul, Turkey

**Keywords:** additive manufacturing, photopolymer, strain sensor, soft application, stereolithography, 3D printing

## Abstract

As a result of the developments in additive manufacturing (AM) technology, 3D printing is transforming from a method used only in rapid prototyping to a technique used to produce large-scale equipment. This study presents the fabrication and experimental studies of a 3D-printed strain sensor that can be used directly in soft applications. Photopolymer-based conductive and flexible ultraviolet (UV) resin materials are used in the fabrication of the sensor. A Stereolithography (SLA)-based printer is preferred for 3D fabrication. The bottom base of the sensor, which consists of two parts, is produced from flexible UV resin, while the channels that should be conductive are produced from conductive UV resin. In total, a strain sensor with a thickness of 2 mm was produced. Experimental studies were carried out under loading and unloading conditions to observe the hysteresis effect of the sensor. The results showed a close linear relationship between the strain sensor and the measured resistance value. In addition, tensile test specimens were produced to observe the behavior of conductive and non-conductive materials. The tensile strength values obtained from the test results will provide information about the sensor placement. In addition, the flexible structure of the strain sensor will ensure its usability in many soft applications.

## 1. Introduction

The AM method has been widely used in recent years. Systems designed in 3D with a computer are produced directly with 3D printers using this method. Nowadays, it is possible to produce many real-time systems with the AM technique. Medicine [1,2], military applications [3,4], robotics [5,6,7], architecture [8,9], industrial manufacturing [10], textiles [11,12], medicine [13,14], aerospace, space [15,16], and food [17,18] are some of these systems. In addition, AM is preferred for fabrication prototypes or mass manufacturing with articulated manufacturing. AM is generally used for prototypes in fields such as medicine and architecture, and for mass production in fields such as industry.

Sensors and actuator systems in macro [19], micro [20,21], and nano [22] sizes can be fabricated with AM. Different 3D printing techniques have been developed for the fabrication of these systems. Manufacturers prefer these techniques according to the dimensions of the applications to be made. Fused Deposition Modeling (FDM) [23,24], SLA [25,26], Polyjet [27], Selective Laser Sintering (SLS) [28], Inkjet Printing [29], Digital Light Processing (DLP) [30], and Two-Photon Polymerization (TPP) [31,32] are the most widely used 3D printing techniques today. In particular, TPP, SLA, and DLP techniques were found to be superior to other methods in the fabrication of micro and nano systems. In recent years, depending on the technological developments related to polymerization systems, the TPP can be fabricated up to the nano level [33]. Due to its common usage area and cost factors, the FDM is preferred in macro applications and non-sensitive systems. The SLA method is based on the principle of hardening certain regions of the photopolymer resin layer, which is liquid at room temperature. This method has many advantages compared to other methods, such as low material consumption, low cost, and high surface quality. The best feature of SLA is its excellent surface quality. In addition, it offers the possibility of parts in end-product quality with its high resolution and strength value. Functional prototyping is an ideal manufacturing technology for patterned structures, molding, and tooling applications. In addition, it is used in applications, such as artificial tissues, microfluidics, biomedical devices, and high electrical capacitors [34,35,36].

When we look at the literature, the fabrication of the strain sensor has been produced with 3D fabrication techniques, such as DLP, FDM, and Inkjet. In a study, flexible material was used in the strain sensor. According to the usage areas, signal data were taken from this sensor and analyzed according to different techniques. It has been stated that the sensor produced with Inkjet is of higher quality in terms of homogeneity and roughness compared to the FDM [37]. In another study, AM of strain sensors with different designs was made with FDM. Experimental analyses of the strain sensor produced due to environmental changes were obtained. Proto-Pasta conductive Polylactic acid (PLA) material was preferred for conductivity. In experimental studies, the viscoelastic properties of the strain sensor were also investigated. For this reason, standard tensile tests of the strain sensor using both conductive PLA and non-conductive PLA material were performed and compared [38]. The fabrication of the strain sensor was carried out using piezoelectric material, and the dynamic performances of the strain sensor produced using the FDM were obtained. The piezoelectric properties of the sensor, its temperature performance, nonlinear states, and behavior at certain frequency values were also investigated [39].

In addition to conductive PLA and piezoelectric materials, different types of nano or micro materials are used in the fabrication of strain sensors. Cheng et al. fabricated strain sensors by the surface assembly of nanoparticles [40]. As a result of experimental studies, it has been found that the strain-sensing properties are highly modifiable at both molecular and nanoscale levels. Huang et al. produced highly sensitive strain sensors based on molecules—gold nanoparticle networks for high-resolution human pulse analysis [41]. The combination of particles with binders provides a covalent 3D network that can be placed directly on prepatterned flexible supports exposing interlocking gold electrodes. Maturi et al. worked on the surface stabilization of ultra-fine gold nanowires (AuNWs) for capacitive sensors in flexible electronics [42]. AuNWs have shown great potential for applications in flexible electronics. Zhang et al. worked on a flexible strain sensor based on a carbon black/silver nanoparticle composite for human motion detection [43]. In this study, carbon black and silver nanoparticles as sensing materials and a thermoplastic polyurethane compound as a matrix were preferred to produce a flexible strain sensor.

The aim of this study is the design and fabrication of a strain sensor for soft applications with SLA, one of the AM techniques. Photopolymer-based photoconductive and flexible UV resins were used as materials for the fabrication of the strain sensor. These resins have a photopolymerizable structure and are both electrically conductive and flexible materials. The operating characteristics of the produced sensor under loading and unloading conditions were observed. In addition, tensile test specimens were produced to observe the behavior of conductive and non-conductive materials. The fabrication of the strain sensor using an SLA-based 3D printer has shown the originality of this study and it is thought that it will contribute to the literature in this respect. In addition, the flexible structure of the strain sensor will ensure its usability in soft applications.

The rest of paper is organized as follows. In the Section 2, the design criteria of the strain sensor and the tensile test specimen are given. Then, the UV material used in production is introduced. Finally, the Stereolithography and experimental setup is explained. In the Section 3, 3D printing processes of strain sensor and tensile test specimens are explained. Then the applied tests, the results of these tests, and the discussion are given. Finally, in the Section 4, the analyses of the data obtained as a result of this study are explained and interpreted. 

## 2. Materials and Methods

### 2.1. Materials

In the AM process, it is possible to create 3D structures using plastic or polymer-based materials, depending on the manufacturing technique used. In this study, photopolymer-based photoconductive and flexible UV resins were used to produce strain sensor by SLA. These resins have a photopolymerizable structure. They are also electrically conductive and flexible materials. Non-conductive conventional UV resin was used to compare the material properties of the tensile test specimens. 

Two types of UV resins, both conductive and non-conductive, were used in the fabrication of the tensile test specimens. In the fabrication of the strain sensor, a conductive UV resin is used for conductive traces, while UV resin with a stretching feature is used for the flexible base. Anycubic brand resin was used as the conventional UV resin material (Anycubic, Shenzhen, China), which does not have conductivity and stretching properties. This resin is a professional 3D printing resin with a low odor, is easy to use, and has high dimensional consistency. 

Some technical properties of conventional UV resin, such as hardness, viscosity, tensile strength, and elongation, are 79 D, 552 mpa.s, 23.4 MPa, and 14.2%, respectively. Wanhao brand photoconductive resin (Wanhao, Jinhua, China) was used as the UV resin material with conductivity. This material is obtained by adding conductive nanoparticles to conventional UV resin. Some technical properties of conductive UV resin, such as hardness, thermal conductivity, electrical resistivity, and thermal expansion, are given in Table 1. 

Finally, Anycubic brand flexible resin (Anycubic, Shenzhen, China) was used as the UV resin material with a stretching feature. This material exhibits good strength and elongation, as well as flexibility. It also provides high accuracy, smooth surface, and low shrinkage rate for 3D printed parts. Some technical properties of flexible UV resin, such as tensile strength, elongation at break, flexibility, and bending strength, are 40 MPa, 40%, 1000 MPa, and 55 MPa, respectively.

### 2.2. Design of Strain Sensor

Strain refers to the rate at which a material under load changes its shape compared to the state before the load is applied. There are four different strain methods in the literature: axial, shear, torsion, and bending. One of these methods is preferred according to the type of material and application area. In this study, the tests of the strain sensor were carried out using the axial strain method. Since the strain sensor is resistance based, the resistance value in the sensor varies in proportion to the strain. Thus, the tensile properties of the sensor can be determined.

**Figure 1:**
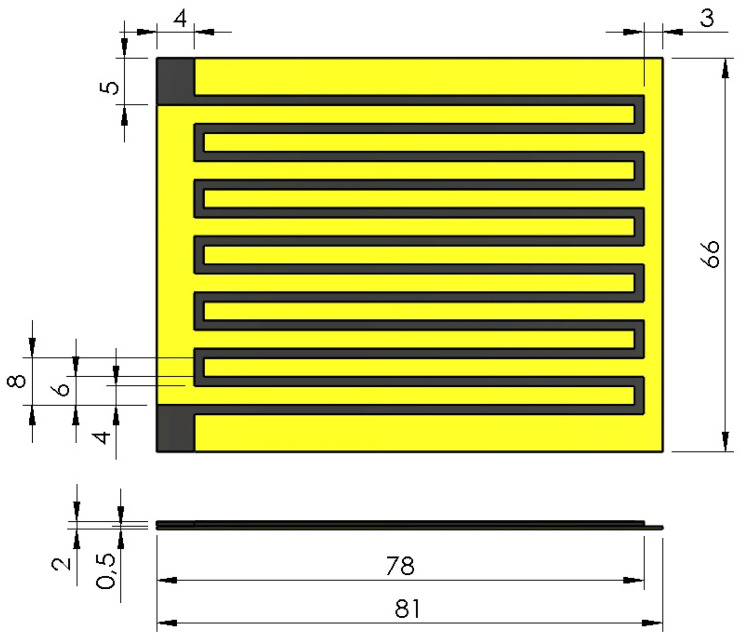
The design of the 3D-printed strain sensor with flexible UV resin body (yellow) and conductive UV Resin tracks (black).

The design of a flexible and conductive 3D-printed strain sensor is shown in Figure 1. Dimensions in the design are given in mm. The sensor body is printed from flexible UV resin material, while detection traces are printed from conductive UV resin material. The 3D fabricated structures replace a conventional strain sensor’s thin conductive metallic foil or wire structure. Thus, while the flexible body of the sensor bends under the applied forces, the thin conductive traces embedded in it will follow the bending and cause a change in resistance.

Looking at the cross-section of the designed strain sensor, a flexible material layer with a thickness of 0.5 mm is placed on the bottom of the conductive layer. Thus, the total thickness of the sensor is 2 mm, and the thickness of the conductive detection traces is only 1.5 mm. These design dimensions allow the sensor to remain flexible and not limit the integration between the base and conductive tracks. In addition, it is designed to include flexible base conductor sections to facilitate wiring from the ends of the conductor rails and to obtain products with dimensions suitable for the tensile tester.

The resistance of a conductive material is calculated as in Equation (1), where R is the resistance, ρ is the resistivity, l is the length, and A is the cross-sectional area. The strain that occurs when the strain sensor is subjected to axial load will reduce the cross-sectional area and increase the length. Thus, the resistance value at the conductive terminals with dimensions of 4mm × 5mm will increase. The fractional change in resistance value is proportional to the strain as shown in Equation (2). The gauge factor, GF, describes the sensor sensitivity to the strain ε.

(1) R=ρl/A

(2) ΔR/R=GF×ε

**Figure 2:**
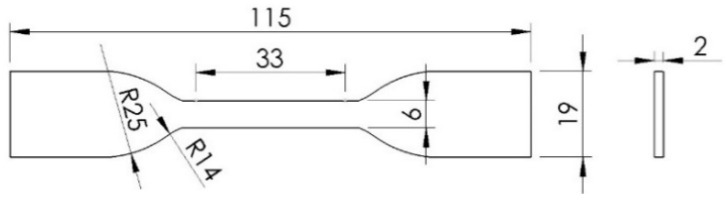
The specimen dimensions (mm) for the tensile test.

A dog-bone-shaped tensile specimen was designed to understand the properties of both the conductive UV resin material used as well as the fabrication of the strain sensor. The dog bone dimensions were followed by the ASTM D638-14 standard [44]. The technical drawings and dimensions of the test specimens that were designed considering this standard are shown in Figure 2. The thickness of the samples was determined as 2 mm.

### 2.3. Stereolithography Method

**Figure 3:**
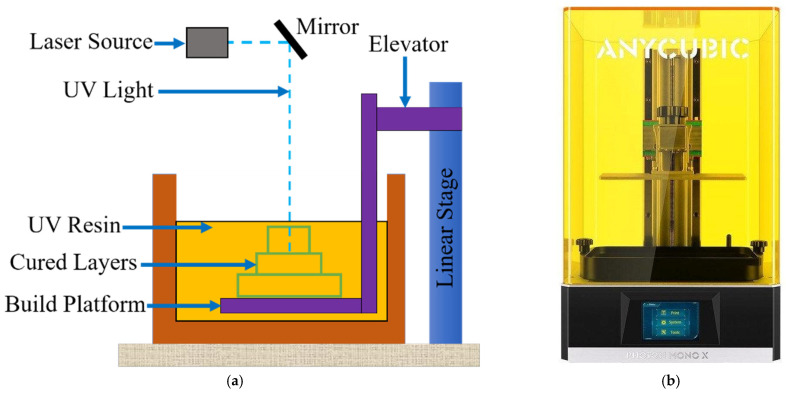
Schematic of SLA system (**a**) SLA-based 3D printing process (**b**) SLA-based 3D printer.

The SLA fabrication procedure illustration is shown in Figure 3a. In this procedure, photopolymer liquid is used. It starts with the photopolymer resin being filled into a reservoir and a moving platform just below the liquid resin surface. Predetermined sections with computer control perform solidification on the liquid resin surface with a UV laser. In this method, layer thicknesses vary between approximately 0.025–0.5 mm. With the completion of each layer, the platform goes down as much as the layer thickness. The remaining volume is covered with liquid photopolymer with the help of a vacuum knife, and the new layer is solidified again in the same way. Thanks to the stickiness of the resin used in this method, the layers easily adhere to each other. During the construction of the layers, supports are inserted under the part to prevent movement. Post-process supports are separated from the main part. Photopolymer resins specific to this method are used as the main material in SLA technology. In some materials, additional curing may be required according to the desired properties, and they are subjected to secondary treatment in the UV bath. Post-fabrication parts are cleaned and fired.

The 3D printer device used to produce the strain sensor with the SLA technique is Photon Mono X (Anycubic, Shenzhen, China). The image of the printer used is given in Figure 3b. This printer has an XY resolution of 0.050 mm (3840 × 2400), a layer resolution of 0.01–0.2 mm, and a dynamic Z resolution of 0.01–0.15 mm. It can also print at a maximum speed of 60 mm/h. The laser source used in the printer is equipped with a 405 nm laser, and the maximum building size is 192 × 120 × 245 mm. The Anycubic printer is built with LCD-based SLA printing technology and uses a 4K Monochrome LCD as the light source. The life of this light source is four times longer than a color LCD. The printer can automatically stop the printer after opening the top cover, which blocks 99.5% of the UV light.

### 2.4. Experimental Setup

This section explains the AM process of strain sensor and tensile specimens. For the production phase with a 3D printer, a 3D solid model of the product is created in a computer environment. The completed computer-aided design (CAD) file is saved in Standard Triangle Language (STL) format. This file is then transferred to the slicer software. In this study, Anycubic Photon Workshop software was used for slicing. This software defines the printing parameters (Table 2), and the slicing process is completed. Here, the amount of material that the model will consume and the production time are shown. The production file is created through the GCODE file obtained at the end of the slice. This file translates the model into a language the 3D printer can understand. Finally, the calibration processes for the printer are completed, and the 3D printer is ready for production. Using an SLA-based 3D printer, this process is repeated for each part from start to finish.

Our primary goal in producing the strain sensor is to optimize the sensor design and 3D printer settings so that the thinnest functional sensor can be printed. The thin cross-sectional area of the conductor rails will ensure that they remain functional as strain sensors when bent. Thus, the sensor becomes flexible at the desired level, and flexibility can be maintained. In addition, the size of the detection traces must be determined by considering the resolution of the SLA printer so that the sensor traces can be successfully printed without being thick.

Tensile tests were applied to determine the elastic stretch range of the produced strain sensor and to determine the load at the plastic deformation point. Experimental work of the sensor was carried out at a tensile speed of 0.005 mm/s until it broke. In addition, a tensile test was applied to the strain sensor at elastic points to determine the relationship between strain and the resistance of the 3D printed trace. For this test, a constant load was applied to the sensor in increments of 40 N for 25 s. During the experiment, strain, load, and resistance values were recorded, and gradually applied load was released.

Experimental studies for the tensile test were implemented with the ZwickRoell tensile testing machine. To examine the properties of the conductive UV resin and non-conductive UV resin material used in the production of the sensor, tensile tests of the samples produced in the shape of a dog bone were also performed. Eight specimens were tested at a constant strain rate of 0.01 mm/s, respectively.

## 3. Experimental Results

### 3.1. 3D Printing Process and Testing

**Figure 4:**
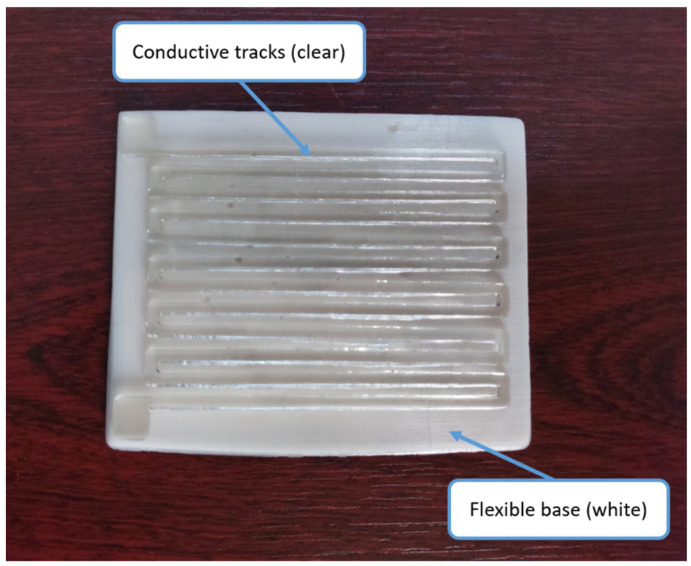
Strain sensor fabricated in an SLA-based 3D printer.

By considering the design criteria mentioned above and following the print parameter settings in Table 2, the functional structure of the strain sensor can be printed consistently. The image of the strain sensor produced in the SLA-based 3D printer is given in Figure 4. The bottom layer of the sensor, which consists of two parts, is produced with 0.5 mm thick flexible UV resin, while conductive detection traces are produced with 1.5 mm thick conductive UV resin. Then, the flexible and conductive parts were bonded with epoxy. When we completely bent the strain sensor sample after production, an average of 3 kΩ change was observed in the resistance value. As expected, the overall change in resistance for the sensor depends on the dimensions of the conductive traces.

**Figure 5:**
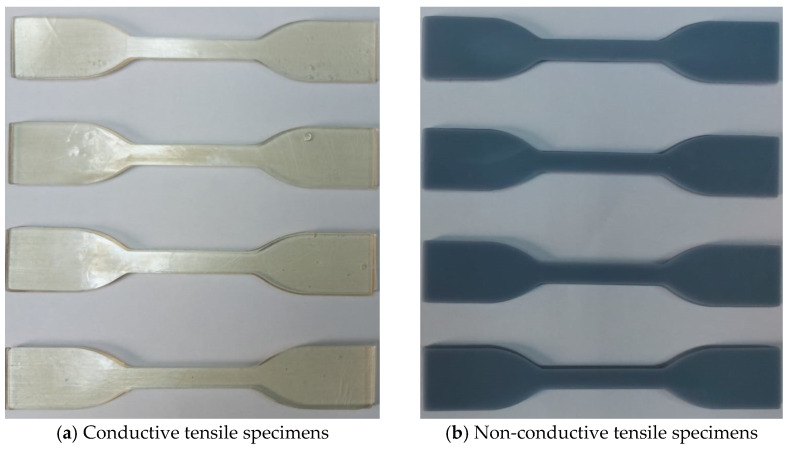
Conductive and non-conductive tensile test specimens fabricated with an SLA-based 3D printer in ASTM D638 standards.

To observe the properties of the conductive UV resin material used after the production of the strain sensor, dog-bone-shaped tensile test specimens were produced. Samples were also produced with a non-conductive conventional UV resin material to compare the conductive tensile samples. Tensile test samples produced in an SLA-based 3D printer in ASTM D638 standards are given in Figure 5. Fabrication parameters are shown in Table 2. Considering these parameters, eight tensile test specimens were produced, four from conductive UV resin, and four from classical UV resin.

### 3.2. Results

**Figure 6:**
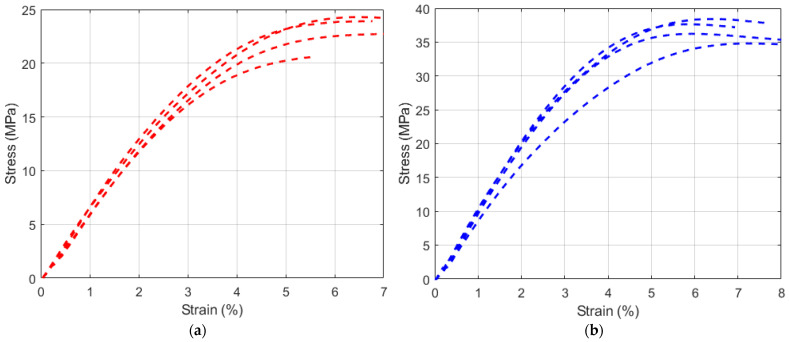
Tensile strength results of UV resin samples (**a**) Conductive UV resin (red) (**b**) Non-conductive UV resin (blue).

Stress-strain results of dog bone pull samples in ASTM D368 standard are given in Figure 6. It has been determined that the conductive UV resin material has a lower strength value than the non-conductive conventional UV resin. In contrast, the average strength value of the conductive samples was 23.76 MPa, measured as 35.23 MPa for the non-conductive samples. It is observed that the strength values of the samples produced with conductive UV resin are reduced by about 30%. It is also seen that non-conductive samples have better mechanical properties when compared to conductive samples. This is because conductive samples have lower mechanical strength due to weak interlayer bonds.

**Figure 7:**
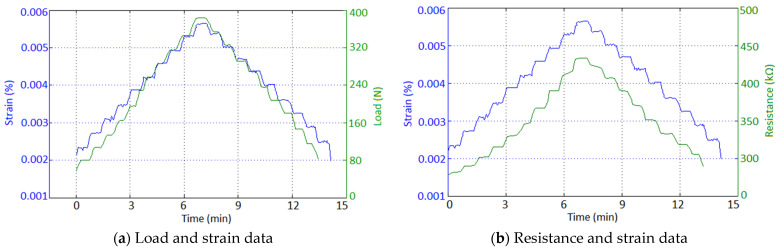
Load-strain and resistance-strain behavior of strain sensor for loading and unloading test.

After the analysis of the tensile test specimens, the mechanical behavior of the strain sensor was observed. Figure 7a demonstrates the load and strain against time for the strain sensor testing. It can be seen that under constant loads, there is no significant increase in strain due to plastic deformation. This indicates that a load of 400 N is within the elastic range of the sample.

Resistance and strain data for the strain sensor loading and unloading test are demonstrated in Figure 7b. The results show a linear relationship between elongation and resistance for the 3D-printed strain sensor. There is a small level of hysteresis observed in the resistance, with a 7.12% increase in resistance between the starting and finishing resistance of the test. During the unloading of the strain sensor, a lagging of resistance was observed relative to the strain measurement. This is thought to be due to the viscoelastic structure of the UV resin.

### 3.3. Discussion

The characterization of the strain sensor has been successfully done with experimental studies. First, tensile test experiments were carried out to observe the difference between conductive and non-conductive UV resin. The results showed that the strength value decreased by about 30% in conductive UV resin samples. In line with these results, improvements and optimizations were made in the production of the strain sensor by understanding the properties of the conductive material.

The relationship between the resistance and strain on the strain sensor was investigated. The results showed a close linear relationship between strain and resistance. This linearity proves the usability of the sensor. Due to the viscoelastic nature of the UV resin, it was observed that the measured resistance values lagged behind the strain values. Although this situation is unfavorable for fast measurements, it is not a problem as strain from tensile tests occurs over a long period.

The glass transition temperature of the UV resin material used in the fabrication of the strain sensor is 104 °C. This value is relatively high compared to materials, such as ABS and PLA, used in other 3D printers. This will prevent the sensor from deforming at high temperatures. Furthermore, since the water absorption property of the resin material is very low, the change in resistance will not occur much. Due to such advantages, the strain sensor produced with flexible and conductive UV resin can be easily applied in soft applications. 

## 4. Conclusions

This study presents the fabrication and experimental studies of a 3D-printed strain sensor that can be used in real-time applications by utilizing AM. An SLA-based 3D printer was used to produce the sensor. Experimental studies were implemented under loading and unloading conditions to observe the hysteresis effect of the sensor. In the tests performed, it was observed that there is a close linear relationship between resistance and strain. In addition to the experimental studies of the strain sensor, tensile tests were performed with both conductive and non-conductive UV resin to understand the material properties and calibrate the sensor. It has been determined that the conductive UV resin material has a lower strength value than the non-conductive conventional UV resin. While the average strength value of the conductive samples was 23.76 MPa, this value was measured as 35.23 MPa for the non-conductive samples. Due to advantages such as high glass temperature and low water permeability, the strain sensor produced with flexible and conductive UV resin can be easily applied in soft applications. The fabrication of the strain sensor using an SLA-based 3D printer has shown the originality of this study and it is thought that it will contribute to the literature in this respect.

## Figures and Tables

**Table 1:** The basic features of the conductive resin.

Elemental Analysis of Resin				
Carbon (at.%)	Hydrogen (at.%)	Nitrogen (at.%)	Oxygen (at.%)	Sulfur (at.%)
20.52	10.24	3.25	15.24	5.18
**Physical and Mechanical Features**				
Density (liquid) g/$\left({\mathrm{cm}}^{3}\right)$	Density (solid) g/$\left({\mathrm{cm}}^{3}\right)$	Young’s Modulus (MPa)	Hardness (MPa)	Tensile Strength
1.65	1.65	375	120	12
**Thermal and Electric Features**				
Thermal Conductivity $\;\left(W/m{}^{\circ}C\right)$	Thermal Expansion $\left(µm/{}^{\circ}C\right)$	Temperature Coefficient $\left(\mathrm{ppm}/{}^{\circ}C\right)$	Electrical Resistivity $\left(\Omega µm\right)$	Relative Permittivity
45	6.2 × 10^−4^	1.75 × 10^−5^	8.5 × 10^−2^	2.5

**Table 2:** Printing parameters of material used for strain sensor fabrication in the SLA 3D printer.

Material	UV Resin	Fill Pattern	Hexagon
Infill Volume (%)	80	Lift Speed (mm/s)	1.5 mm/s
Layer Thickness (mm)	0.04	Off-Time (s)	0.50
Normal Exposure Time (s)	15	Transition Layers	15
Bottom Exposure Time (s)	120	Bottom Layers	25

## Data Availability

Data is contained within the article.
